# Long-Term Maintainable Somatic Embryogenesis System in Alfalfa (*Medicago sativa*) Using Leaf Explants: Embryogenic Sustainability Approach

**DOI:** 10.3390/plants8080278

**Published:** 2019-08-09

**Authors:** Ankush Sangra, Lubana Shahin, Sarwan K. Dhir

**Affiliations:** 1 College of Agriculture, Family Sciences and Technology, Fort Valley State University, Fort Valley, GA 31030, USA; 2 Center for Biotechnology, Department of Plant Sciences, Fort Valley State University, Fort Valley, GA 31030, USA

**Keywords:** somatic embryogenesis, callus, alfalfa, embryogenic sustainability, scanning electron microscopy, subculturing, embryo maturation

## Abstract

Alfalfa (*Medicago sativa*) is one of the most important forage legume crops because of its mass production and high feeding value. It originated in Asia and is one of the most ancient plants cultivated throughout the world as a fodder. Despite the well-studied somatic embryogenesis of alfalfa, there is a lack of a long-term maintainable somatic embryogenic system. Every time an embryogenic callus culture must be started from new explants, which is laborious, costly and time consuming. In addition to this, endogenous microorganisms present in ex vitro explants of alfalfa can often cause contamination, reducing the efficiency of callus culture. An attempt was made to establish long-term continuous somatic embryogenesis system in alfalfa using cultivar Regen-SY. Nine somatic embryogenesis pathways were studied and evaluated for embryo yield, plant conversion rate and embryogenic sustainability. Somatic embryos passed through the same stages (globular, heart-shaped, torpedo and cotyledonary) as characteristic of the zygotic embryo and secondary somatic embryogenesis was also observed. B5H-B5 system showed the highest embryo yield and plant conversion rate whereas SH4K-BOi2Y system demonstrated the highest embryogenic sustainability and maintained the embryogenic potential even after six subculture cycles. Scanning electron microscopy was applied to study the morphology of the somatic embryos and secondary somatic embryogenesis. Therefore, long-term maintainable somatic embryogenesis system protocol was developed through this study, which will help to enhance and accelerate the alfalfa biotechnology research.

## 1. Introduction

Alfalfa is a perennial, flowering member of the Fabaceae family that originated in Asia and is cultivated throughout the world as a fodder [1]. It is an important forage crop because of its mass production and high nutritional value. Alfalfa (*Medicago sativa*) hybrid Regen-SY was released in 1989. It was produced using first generation self-parents from Regen-S (*M. sativa*) and Regen-Y (*Medicago falcata*) research cultivars. Alfalfa Regen-SY was released to introduce improved regeneration traits [2]. Alfalfa is a legume that forms a symbiotic relationship with the bacterium *Sinorhizobium meliloti I*, which fixes atmospheric nitrogen [3].

Somatic embryogenesis is a regeneration system in which somatic cells are induced under in vitro conditions to acquire embryogenic potential. Somatic embryos resemble zygotic embryos and pass through subsequent stages characteristic of zygotic embryos [4]. Somatic embryogenesis is the main pathway of regeneration in many plant species [5] and provides an important tool that forms the basis of the genetic modifications in plants [6]. Somatic cells contain the entire set of information necessary to create and complete a functional plant [7]. Somatic embryos were first reported in carrot and since then it has been reported in many plants [8]. Unlike zygotic embryos, somatic embryos are devoid of endosperm, which provides nutrients and plant growth regulators to the zygotic embryos. Therefore, adding plant growth regulators and nutrients in the culture medium could be helpful to the somatic embryos [9].

Studies have indicated that in vitro response of alfalfa is highly genotype dependent [10,11]. Antassov and Brown (1984) [11] reported the induction of callus using cotyledon, hypocotyl and leaf-derived alfalfa explants on B5 medium supplemented with plant growth regulators [12]. Somatic embryogenesis has been induced on B5h and SH4K media in alfalfa genotype N4 [13,14]. Embryos started forming in B5h medium while the explants were still on the induction medium. In case of SH4K medium, embryos did not form until explants were transferred to growth regulator free medium [15].

Despite having a well-studied somatic embryogenesis system, alfalfa has remained conspicuous among many plants that lack maintainable embryogenic cultures. There is no long-term continuous somatic embryogenesis culture protocol for alfalfa. Alfalfa callus is known to lose their capability to form embryos after two to three subcultures. So far, very little efforts and progress have been made to establish a maintainable long-term continuous embryogenic culture. Conventionally, secondary somatic embryogenesis has been used to prolong the somatic embryogenesis of alfalfa [16]. A callus culture of alfalfa somatic embryogenesis is required to be started with new explant every time, which is laborious, costly and time consuming. In addition, the presence of endogenous microorganisms in the ex vitro explants of alfalfa results in a high degree of contamination, which further decreases the efficiency of new cultures [15,17].

Lack of continuous embryogenic culture is a major hindrance towards the research progress and commercial applications of the alfalfa somatic embryos. Many research methodologies such as genetic transformation and synthetic seed technology require continuous mass production of somatic embryos. Therefore, there is a broad interest in studying the mechanism underlying the long-term maintainable embryogenesis and establishing protocol for the same.

## 2. Results

### 2.1. Callus Induction

**Figure 1:**
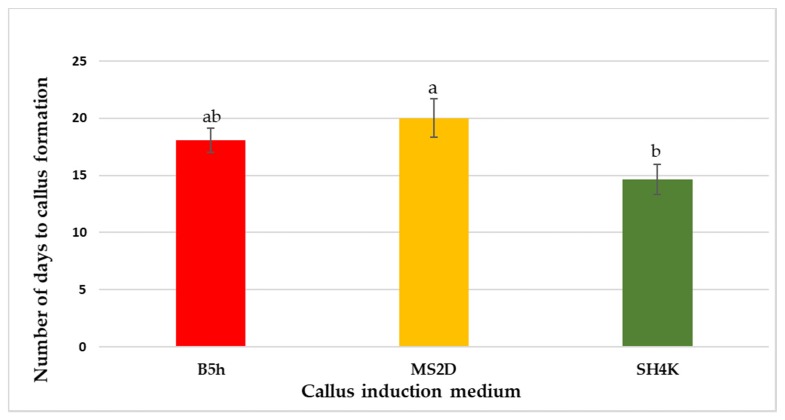
Number of days to callus formation on a different callus induction medium. Different letters indicate a significance difference according to the Tukey Kramer honestly significance difference (HSD) test at *p* ≤ 0.05 and values represent mean ± SD (*n* = 30).

A combination of 2,4-dichlorophenoxyacetic acid (2,4-D) and kinetin was used to induce callus formation in all the somatic embryogenesis systems. Callus formation started with curling of leaf layer explants. Different patterns of curling were observed (inward, outward, upward, etc.), therefore callus formed was of different shapes. Callus induced on B5h and MS2D medium was greenish or yellowish in color whereas callus induced on SH4K medium was yellowish or white in color. Time taken for the complete callus formation also varied on each callus induction medium (Figure 1).

### 2.2. Somatic Embryogenesis and Its Phases

**Figure 2:**
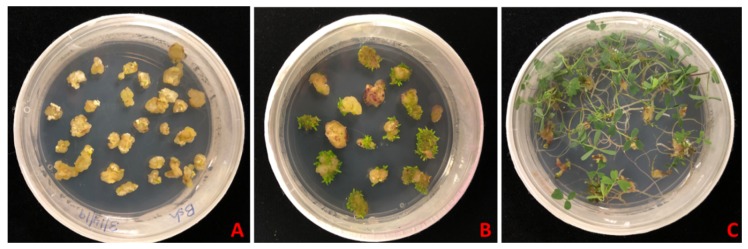
Three phases of somatic embryogenesis observed on the B5h-B5 system. (**A**) Callus induction, (**B**) somatic embryogenesis and maturation and (**C**) embryo germination.

**Figure 3:**
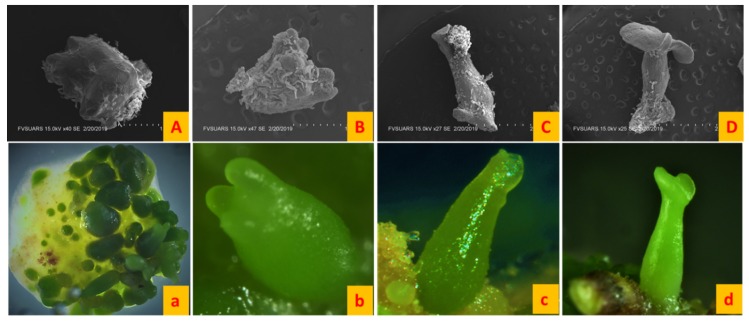
Different stages of somatic embryogenesis as seen under the light microscope (**a**–**d**) and scanning electron microscope (**A**–**D**). (**A**,**a**) Globular shaped, (**B**,**b**) heart shaped, (**C**,**c**) torpedo shaped and (**D**,**d**) cotyledonary staged. For all SEM images (**A**,**B**) bar = 1.00 mm, (**C**,**D**) bar = 2.00 mm and all light microscope images at 12× magnification. All stages were taken from the SH4K-BOi2Y system.

Somatic embryogenesis was studied on nine systems and each system consisted of three mediums: The callus induction medium, embryo development and maturation (EDM) medium and embryo germination medium. Leaf layer explants were placed on the callus induction medium and leaf layer curling was observed within three days of the inoculation of explants in all the systems. Leaf curling showed different patterns and callus formation began from the margins of explants, later on converting whole explant into callus (Figure 2A). After three weeks, the callus was placed on the EDM medium. Induction of somatic embryos started within three to four days of the transfer and embryos were allowed to mature for three weeks (Figure 2B). After the maturation period, embryos were germinated in the germination medium in 100 mm × 25 mm petri dishes (PhytoTechnology laboratories). Approximately 15 somatic embryos were germinated in one petri dish (Figure 2C). Once the embryos formed a plantlet, each plantlet was then transferred to individual culture jar (PhytoTechnology laboratories). Figure 2 shows the three phases of somatic embryogenesis. The first phase is callus induction and 100% callusing can be seen (Figure 2A). The second phase is embryo formation and maturation in which embryos form and pass through different stages to get matured (Figure 2B). The third phase is embryo germination in which embryos germinate to form plantlets with root and shoots (Figure 2C). Somatic embryos passed through all the stages characteristic of zygotic embryos (Figure 3).

#### 2.2.1. Embryo Development and Maturation

**Figure 4:**
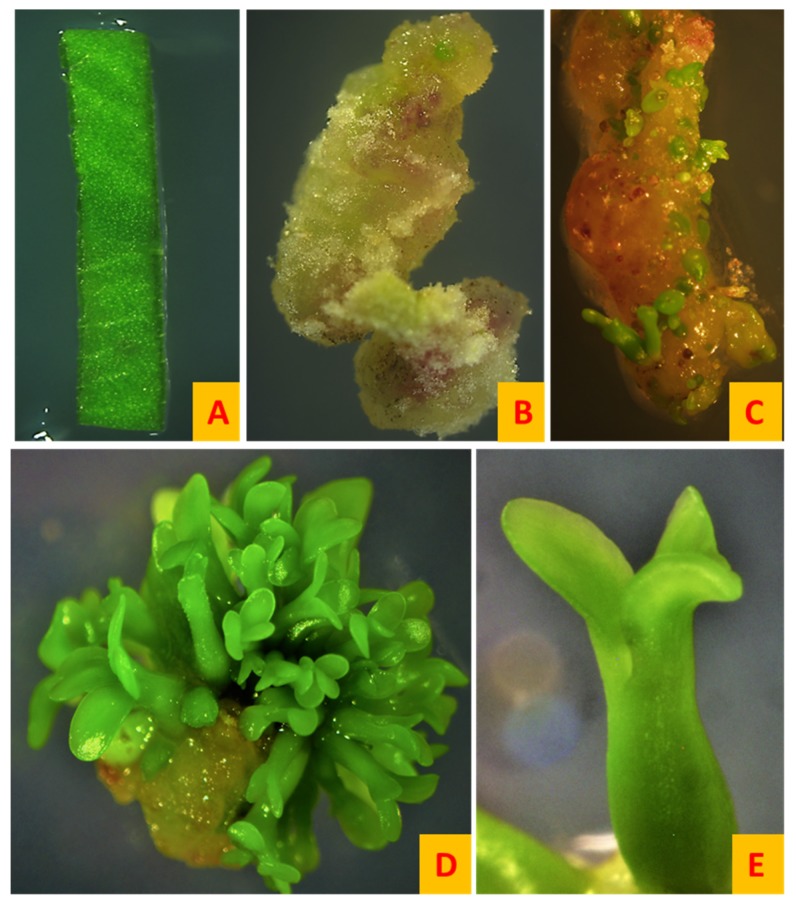
Somatic embryogenesis on the B5h-B5 system. (**A**) Inoculation of the leaf layer explant on the B5h medium, (**B**) complete callus formation after 18 d on the B5h medium, (**C**) induction of somatic embryos after seven days on the B5 medium, (**D**) synchronous culture of mature somatic embryos after 21 days on the B5 medium and (**E**) mature cotyledonary stage embryo ready to be harvested after 21 days on the embryo maturation medium. All images were at 8× magnification except Image E (24×).

**Figure 5:**
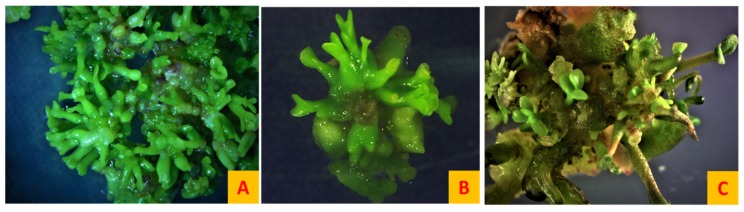
Secondary somatic embryogenesis. (**A**) Early secondary somatic embryogenesis where dense culture of mature somatic embryos giving rise to new globular embryos from the surface of primary embryos in B5h-B5 system. (**B**,**C**) Late secondary somatic embryogenesis where cotyledonary and torpedo staged secondary somatic embryos arising from the primary somatic embryos can be seen in the SH4K-BOi2Y system. All images were at 8× magnification.

Most of the embryogenic cultures started as an asynchronous culture (Figure 4C) but they attained synchrony as they reached the maturation stage (Figure 4D) Once the callus was transferred to the EDM medium, the number of somatic embryos formed were recorded at 7, 14 and 21 days. While counting the embryos only torpedo and cotyledonary staged embryos were taken into consideration. Table 1 shows the somatic embryo yield of each system after 7, 14 and 21 days. Somatic embryo formation was observed in all systems except the SH4K-MS system. Secondary somatic embryogenesis was also observed in many systems in which secondary somatic embryos arose from the surface of the primary embryos (Figure 5).

#### 2.2.2. Comparison of Somatic Embryogenesis in Different Systems

All the systems except the SH4K-MS system resulted in somatic embryogenesis. There were morphological differences between the callus induced on a different callus induction medium as the callus induced on the MS2D and B5h medium was greenish or yellowish in color whereas the callus induced on SH4K was white or yellowish in color. The callus induced on the SH4k medium was softer than the MS2D and B5h callus. In all systems embryo induction started three to four days after transfer to the EDM medium. In most systems rapid embryo formation was observed between 10 and 20 d after transfer to the EDM medium. The B5h-B5 system was most yielding (48.85 embryos per explant) where as SH4K-MS did not result in any formation of somatic embryos. There was an initial induction of somatic embryos on SH4K-MS system, but these somatic embryos died just after induction. As the SH4K callus formed embryos on the other EDM media (B5, BOi2Y), therefore it can be hypothesized that the composition of the MS medium as an EDM medium is not suitable for the SH4K callus. The B5h callus started forming globular embryos while on the callus induction medium but this was not true for the MS2D and SH4K callus. By comparing all systems, it was found that all three callus induction media caused embryogenic callus induction. As far as EDM medium is concerned the MS medium as the EDM medium worked well only with the MS2D callus as in other systems it either resulted in low yield (B5h-MS) or no embryo formation (SH4K-MS). Out of the three EDM media used B5 worked well in the all the systems resulting in high embryo yield in all the systems. The B5h-B5 system was the most prolific as it showed high yield and rigorous secondary somatic embryogenesis if left on EDM media after maturation of primary somatic embryos. Some systems such as B5h-MS and MS2D-BOi2Y were prone to the premature embryo death. In the MS2D-BOi2Y system, the embryo yield was more after 14 days on the EDM medium as compared to 21 days because many somatic embryos in this system died prematurely (Table 1).

#### 2.2.3. Embryo Germination and Acclimatization

**Figure 6:**
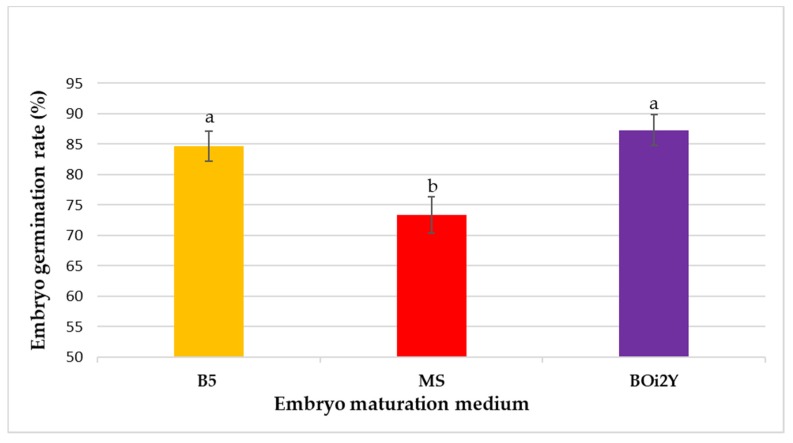
Embryo germination rate on the MMS medium after being transferred from a different maturation medium. Values represent mean ± SD (*n* = 30) and different letters indicate a significant difference according to the Tukey Kramer HSD test at *p* ≤ 0.05.

**Figure 7:**
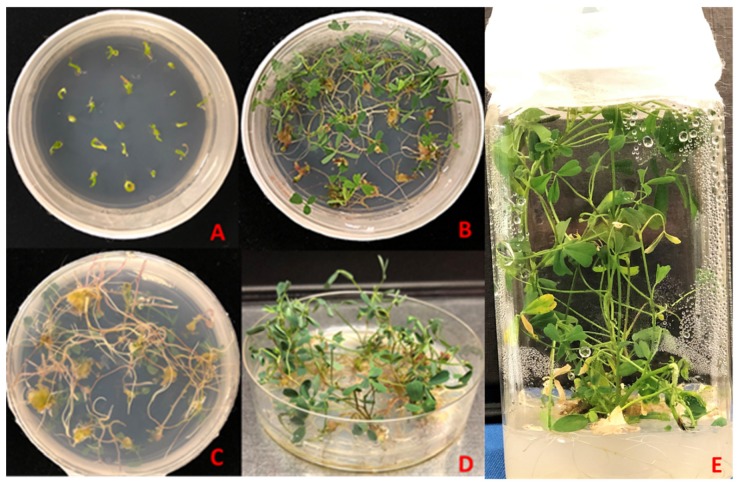
Embryo germination and rooting. (**A**) Mature embryos picked and germinated separately, (**B**) embryos forming plantlets after germination, (**C**) root formation after somatic embryo germination, (**D**) plantlets ready to be transferred and (**E**) an in vitro plant produced from somatic embryo.

Once the embryos matured and reached the cotyledonary stage (Figure 3D), the embryos were picked with the help of sterilized forceps and transferred to the germination medium (MMS) in 100 mm × 25 mm petri dishes containing 30 mL of germination medium. The embryo germination rate was recorded separately for the embryos transferred from different maturation medium (B5, MS and BOi2Y). Once the plantlets reached 3–5 cm in height, they were transferred to culture jars (PhytoTechnology Laboratories). Embryos transferred from the BOi2Y medium showed the highest germination rate whereas embryos transferred from the MS medium resulted in the lowest germination rate (Figure 6). Embryos formed shoots and roots simultaneously (Figure 7). There was no significant difference between the embryo germination rate of embryos matured on B5 and BOi2Y medium but embryos transferred from the MS medium showed a lower germination rate.

For acclimatization, the roots of the plantlets were washed with the sterile distilled water to remove the culture medium and transferred to the magenta boxes containing sterile potting mix with 5 mL of the MSO medium. An acclimatization apparatus was formed by joining two magenta boxes with the connector. After a week, the connector was loosened to reduce humidity and after three weeks the upper box was completely removed. Plantlets were then transferred to the green house and fully fertile plants were recovered.

### 2.3. Embryogenic Sustainability

**Figure 8:**
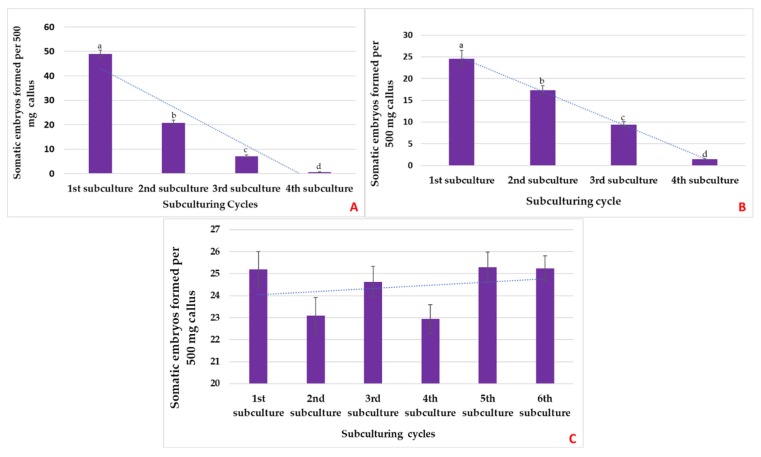
Embryogenic sustainability through subculturing cycles. (**A**) The B5h callus, (**B**) MS2D callus and (**C**) SH4K callus. The B5h callus and MS2D callus both gradually lost embryogenic potential through the subculturing cycles where the SH4K callus retained its embryogenic potential even after six subcultures without showing any significant loss in the embryo yield. Values represent means ± SDs (*n* = 30) and different letters indicate significant differences according to the Tukey Kramer HSD test at *p* ≤ 0.05.

To determine the embryogenic sustainability of the B5h callus, a callus was initiated on the B5h medium and subcultured on the fresh B5h medium after every 18 days. While subculturing 500 mg of callus was transferred to the EDM medium (B5) and the remaining callus was subcultured on a fresh B5h medium. This process was repeated every 18 days and the mature embryos formed on the B5 medium were counted after 21 days. Alfalfa callus cells are known to lose embryogenic potential through the subcultures [16]. It was observed that embryo formation was the highest after the first subculturing showing both primary and secondary somatic embryogenesis. B5h callus gradually lost its embryogenic potential and it showed no somatic embryo induction after fourth subculture (Figure 8A). As the B5h callus went through subcultures, it became brown and hard as compared to the initial callus. Embryogenic sustainability of the MS2D callus was determined by subculturing MS2D callus after every 18 days. While subculturing, 500 mg of callus was transferred to the MS medium and embryos were counted after 21 days. The highest yield of somatic embryos was recorded after the first subculture per 500 mg callus. There was a loss in embryogenic potential with every subculture and after the fourth subculture cycle there was no somatic embryo formation (Figure 8B). The callus became brown and hard with every subculture and lost its embryogenic potential. The MS2D callus showed the same characteristic as that of the B5h callus resulting in a non-embryogenic callus mass after the fourth subculture.

**Figure 9:**
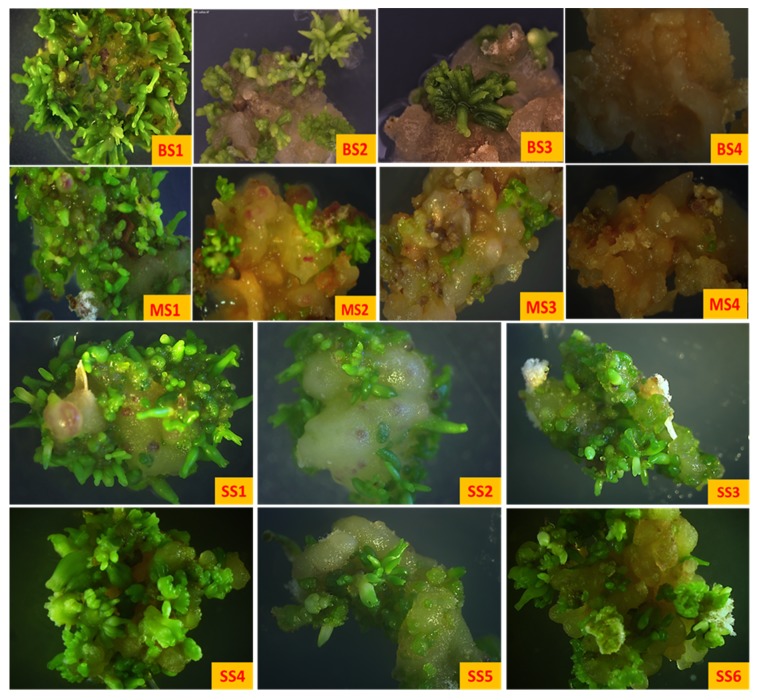
Embryogenic sustainability of the callus through the subculture cycles. (BS1–BS4) Embryogenic sustainability of the B5h callus from the first to fourth subculturing, (MS1–MS4) embryogenic sustainability of the MS2D callus from the first to fourth subculture and (SS1–SS6) embryogenic sustainability of the SH4K callus from first to sixth subculture. All images were taken after 21 days on the EDM medium. All images were at 8× magnification.

Embryogenic sustainability of the SH4K callus was determined by inducing the callus on the SH4K medium. The callus was subcultured every 18 days and while subculturing 500 mg of the callus was transferred to the BOi2Y medium for embryo formation. This process was repeated after every subculture cycle. Observations revealed that the SH4K callus maintained its embryogenic potential through the subculturing cycles and showed constant formation of somatic embryos. The trendline on the graph (Figure 8C) stayed in between 24 and 25, which shows that there was no significant difference in the embryo yield after each subculture. Therefore, it was observed that the SH4K callus was maintainable and it retained its embryogenic potential without showing a significant loss in it. The SH4K callus showed minimum browning through the subcultures and retained its color and softness (Figure 9).

### 2.4. Scanning Electron Microscopy and Morphology of Somatic Embryogenesis

**Figure 10:**
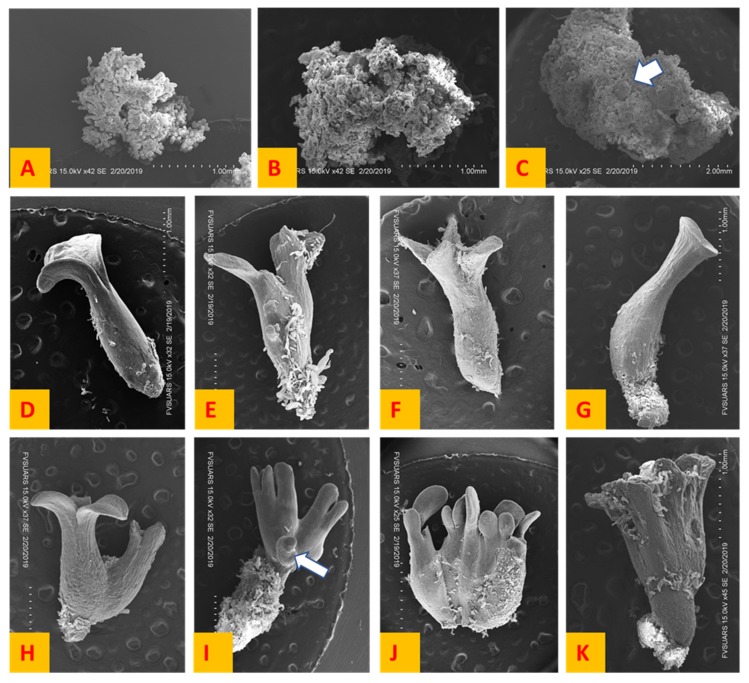
Scanning electron micrographs. (**A**) Loosely arranged disorganized cells in the callus, (**B**) cells getting compactly arranged to form globular embryos, (**C**) formation of globular embryos (indicated by an arrow) among the compactly arranged callus cells, (**D**) monocotyledonous, (**E**) dicotyledonous, (**F**) polycotyledonous, (**G**) long hypocotyl, vestigial cotyledon, (**H**) proximal di-axial fusion, (**I**) di-axial fusion with emergence of a secondary embryo (indicated by an arrow) at the base, (**J**) moderately fasciated and (**K**) gross fasciation.

Scanning electron microscopy was applied to study the morphology and induction of somatic embryogenesis. Scanning electron microscopy was done using Hitachi S-3400N scanning electron microscope. Callus at the initial stages appeared as the loosely arranged elongated cells and after transfer to the EDM medium cells got compactly arranged and globular shaped embryos appeared in between the compactly arranged cells (Figure 10A–C). Figure 10 shows the various kinds of somatic embryos obtained. There was a considerable difference in the mature embryos harvested from the cultures. The size of mature somatic embryos harvested varied from 2 to 6 mm. Based on the variation in the morphology of the somatic embryos obtained, somatic embryos were classified into eight types (Figure 10D–K).

## 3. Discussion

All the somatic embryogenesis systems except the SH4K-MS system produced viable somatic embryos and secondary somatic embryogenesis was observed when cultures were retained on the EDM medium after the maturation of primary embryos. The SH4K-MS system showed the induction of somatic embryos, but these embryos died in two to three days after induction. The initial callus was a mass of loosely arranged nodular cells and callus cells became more compactly arranged as the induction of embryos started. The callus mainly consisted of the long vacuolated suspensor cells and some globular shaped structures were seen, indicating the initial stages of somatic embryo formation (Figure 10A–C). At the early globular stage embryos were oval structures and at the late globular stage they became elongated oval structures [18]. Somatic embryos passed through all stages but sometimes they could skip a stage or two as the initiation of cotyledon formation could be seen at the heart shaped stage (Figure 3b). In the secondary or repetitive somatic embryogenesis, primary embryos gave rise to secondary embryos in culture. Scanning electron microscopy showed loose large vacuolated elongated cells projecting from the surface of the somatic embryos (Figure 10). These cells could be responsible for the secondary somatic embryogenesis in alfalfa. Dos Santos et al. [19] reported that alfalfa somatic embryos arise from the single epidermal cell, but the author did not rule out the possibility of multicellular origin. Studies have shown that the origin of somatic embryos differ from plant to plant. In the *Quercus* species, it has been observed that secondary embryos arise from the root pole of the primary embryos [20], whereas walnuts, cotyledons and hypocotyl give rise to secondary embryos [21]. Secondary somatic embryos arising from the surface of the primary embryos were observed in many systems (Figure 10I). Studies have revealed that secondary somatic embryos have normal development probably because of their single celled origin [22,23]. The hypothesis behind this is that secondary somatic embryos arise from the cells without mutations as cells with mutations are removed by programmed cell death as a result of a cellular mechanism to remove DNA damage [24].

High production of abnormal somatic embryos has been reported in many plant species [25,26]. In our study major somatic embryo abnormalities observed were monocotyledonous, polycotyledons, fasciation and vestigial cotyledons (Figure 10D–K). Abnormalities in somatic embryogenesis have often been associated with physiological disorders and somaclonal variations where mutations or epigenetic changes can influence the process of embryo development, which results in abnormal morphology of the resulting plants [25,27,28]. Auxins are used to induce somatic embryogenesis in many plant species. However, auxin can produce genetic and epigenetic changes in the cells such as mutations and methylation of DNA [29,30]. Thus, auxins have been related to the abnormal somatic embryo formation because at high concentration it can disrupt the normal genetic and physiological processes in the cells [31,32]. In our study, we used auxin (2,4-D) to induce somatic embryogenesis and exposition or accumulation of auxin in the tissue could be another reason for the formation of abnormal embryos.

Somatic embryogenesis induced on the SH4K medium resembled that of conifers and some cereals. In conifers and cereals, the callus induced can be maintained by subculturing on to the medium lacking plant growth regulators [33,34]. Cells are less differentiated in these cultures. In conifers, the embryonal mass tissue maintained by the subculture shows visible embryo heads and suspensor, but these structures never develop into embryos unless they are transferred to the plant growth regulator free medium [34]. The SH4K callus maintained through subculturing showed an embryo like structure but these structures remained juvenile during the subculturing process and only developed into mature somatic embryos when the callus was transferred to the embryo development and maturation medium.

The difference in the morphology of the callus obtained could be because of the difference in the composition of all the media (B5h, MS2D and SH4K) used in the study. As a future prospective to this study, it would be interesting to see whether additional chemicals added to the medium are responsible for making the SH4K callus maintainable or its basal medium composition that makes it maintainable. In addition, studies can be performed in the future on the germination rate of the different types of abnormal embryos obtained. As we observed that SH4K callus could be maintained for a long time, it would be another interesting study to see if there is any correlation between genetic variability of the plants and number of subcultures.

## 4. Materials and Methods

### 4.1. Plant Material and Germination

Alfalfa cultivar Regen-SY germplasm (PI 537440) was supplied in the form of seeds by the Western Regional PI Station through U.S. National Plant Germplasm System. Seeds were surface sterilized by submerging in 70% ethanol for 30 s. After the removal of ethanol, seeds were treated with 20% bleach solution with 0.05% to 0.10% of Tween 20 for 10 min with occasional stirring. Seeds were then rinsed with sterile water three times. Individual seeds were then placed in the magenta GA7 boxes containing MSO medium. Nine seeds per box were inoculated and incubated at 25 °C under lights with photoperiod of 16 h of 60 to 80 µE/m^2^/s.

#### 4.1.1. Embryogenic Callus Induction

Several leaves from well grown, three to four weeks old plants were carefully removed. Leaves were cut into six layers of approximately uniform thickness with the help of sterile scalpel blade. Leaf explants thus prepared were then placed on the callus induction media in a 100 mm × 25 mm Petri dishes (PhytoTechnology Laboratories). B5h, MS2D and SH4K were used as the embryogenic callus induction media. Petri plates were then placed in the growth chamber at 22 to 24 °C under dark conditions in the growth chamber (Percival Scientific Inc.). Dark conditions were created by wrapping aluminum foil around the Petri dishes.

#### 4.1.2. Embryogenesis and Maturation

After three weeks, the callus was transferred to the EDM medium, which is a growth regulator free medium (B5, MS and BOi2Y) to stimulate the formation of embryos.

#### 4.1.3. Germination of Embryos

Once the embryos were formed and reached the cotyledonary stage, they were transferred to the germination medium (MMS) for the conversion to the plantlets. Embryos were picked with the help of sterile forceps and transferred to petri dishes containing the MMS medium. Once they formed plantlets each plantlet was transferred to the culture jar (PhytoTechnology) containing the MSO medium.

#### 4.1.4. Somatic Embryogenesis Systems Studied

We studied nine somatic embryogenesis pathways, which are given in the Table 2. Each pathway consisted of callus induction, embryo development and maturation (EDM) medium and germination medium. The composition of all the media used in the study is given in Table 3.

### 4.2. Evaluation of Embryogenic Sustainability

Leaf layer explants of alfalfa Regen-SY were prepared and placed on a different callus induction medium; B5h, MS2D and SH4K media. After induction of the embryogenic callus, the callus from B5h, MS2D and SH4K was subcultured on the fresh B5h, MS and SH4K respectively. While subculturing, 500 mg of callus was transferred to B5, MS and BOiTY medium and the number of embryos formed were counted and germinated in germination media after 21 days. Subculturing was repeated after 18 days and the number of embryos formed each time was considered as proof of the embryogenic sustainability. The long-term continuous somatic embryogenesis system was identified on the basis of the longevity of the embryogenic potential through subculture cycles.

### 4.3. Scanning Electron Microscopy

Embryogenic calli and somatic embryos of different morphology at different stages were used as a specimen. Primary fixing was done by placing specimen in 2% glutaraldehyde in phosphate buffer saline (PBS) with pH 7.2 for one hour at 25 °C. Samples were then washed with PBS three times for 15 min. For secondary fixing, samples were placed in 1% osmium tetroxide (OsO_4_) in PBS for 1 h at 25 °C. Samples were then washed three times with distilled water for 15 min. Dehydration was done using an ascending series of ethanol (25%, 50%, 75%, 95% and 100%) for 15 min each. Finally, samples were placed in 100% ethanol three times for 15 min. Critical point drying (CPD) was done using Leica EM CPD 300 automated critical point dryer. The critical point for CO_2_ was reached at 35 °C and 1200 psi. Samples were placed in the aluminum stub using double sided carbon tape. Stubs were numbered and descriptions were written. Sputter coating of the gold target was done using Denton Vacuum Desk V sputter coater.

### 4.4. Statistical Analysis

Each experiment was replicated three times. Experimental data were statistically analyzed using analysis of variance (ANOVA). Treatment means were separated by using the Tukey Kramer honestly significance difference (HSD) test at *p* ≤ 0.05. 

## 5. Conclusions

In this study a protocol for long-term maintainable somatic embryogenesis of alfalfa using cultivar Regen-SY was established. This study demonstrated that an embryogenic callus induced on the B5h and MS2D medium was not maintainable and lost its embryogenic potential at the end of the fourth subculture. On the other hand, a callus induced on the SH4K medium was maintainable and it constantly kept forming somatic embryos after every subculture without any significant loss in embryogenic potential. A lack of continuous somatic embryogenesis protocol in alfalfa is a major hindrance to the research. Most of the research methodologies such as synthetic seed technology, genetic engineering and other in vitro improvement methods need continuous supply of somatic embryos. Through this study, we found maintainable callus culture that remained totipotent throughout subcultures and yielded embryos constantly after every subculture. This protocol for the long-term continuous somatic embryogenesis will accelerate and enhance the efficiency of research in alfalfa, particularly genetic transformation and regeneration studies.

## Figures and Tables

**Table 1:** Number of somatic embryos formed at weekly intervals on the embryo development and maturation (EDM) medium in all systems.

S. No.	Somatic Embryogenesis System Name	After 7 Days	After 14 Days	After 21 Days	Secondary Somatic Embryogenesis
1	B5h-B5	6.6 ± 0.4	22.8 ± 0.9	48.9 ± 1.6	Yes
2	B5h-MS	1.9 ± 0.3	4.9 ± 0.3	5.7 ± 0.3	No
3	B5h-BOi2Y	3.3 ± 0.2	7.7 ± 0.4	27.9 ± 1.0	Yes
4	SH4K-B5	3.4 ± 0.19	7.5 ± 0.3	25.2 ± 0.8	No
5	SH4K-MS	0.0	0.0	0.0	No
6	SH4K- BOi2Y	3.4 ± 0.3	9.6 ± 0.5	32.2 ± 1.4	Yes
7	MS2D-B5	5.2 ± 0.3	16.0 ± 1.2	33.6 ± 2.2	Yes
8	MS2D-MS	4.0 ± 0.3	8.6 ± 0.9	18.0 ± 3.1	No
9	MS2D-BOi2Y	2.2 ± 0.3	6.0 ± 0.4	4.9 ± 0.9	No

Each experiment was conducted three times and values represent mean ± SE (*n* = 30).

**Table 2:** Various somatic embryogenesis systems studied with their medium name for different phases.

S. No	Somatic Embryogenesis System Name	Callus Induction Medium	Embryo Development and Maturation (EDM) Medium	Germination Medium
1	B5h-B5	B5h	B5	MMS
2	B5h-MS	B5h	MS	MMS
3	B5h-BOi2Y	B5h	BOi2Y	MMS
4	SH4K-B5	SH4K	B5	MMS
5	SH4K-MS	SH4K	MS	MMS
6	SH4K- BOi2Y	SH4K	BOi2Y	MMS
7	MS2D-B5	MS2D	B5	MMS
8	MS2D-MS	MS2D	MS	MMS
9	MS2D-BOi2Y	MS2D	BOi2Y	MMS

**Table 3:** Composition of the various media used in the study along with additional chemicals.

Medium	Basal Medium	Growth Regulators	Additional Chemicals
B5h	Gamborg B5 basal salt 1968) [12]	4.5 µM 2,4-D, 0.9 µM kinetin	5.1 mM CaCl_2_.2H_2_0, 5.5 mM glutamine, 32.5 µM glutathione, 95.1 µM serine, 7.4 µM adenine, 3% sucrose, 0.25% Gelrite or 0.7% agar
SH4K	Schenk and Hildebrandt (1972) [13]	4.5 µM 2,4-D, 0.9 µM kinetin	25 mM proline, 0.4 mM thioproline, 50 mM potassium sulfate, 0.2% yeast extract, 100 mg/L *m*yo-inositol, 3% sucrose 0.25% Gelrite or 0.7% agar
MMS	Murashige and Skoog basal salt mixture (1962) [35]	--	1 mL/L 1000× Nitsch and Nitsch vitamin solution (Sigma), 0.1 g/L *m*yo-inositol, 3% Sucrose, 0.25% Gelrite or 0.7% agar
MS2D	Murashige and Skoog basal salt mixture (1962) [35]	4.5 µM 2,4-D, 0.9 µM kinetin	3% sucrose, 0.25% Gelrite or 0.7% agar
B5	Gamborg B5 Basal salt mixture [12]	--	5.1 mM CaCl2.2H20, 5.5 mM glutamine, 32.5 µM glutathione, 95.1 µM serine, 7.4 µM adenine, 3% sucrose, 0.25% Gelrite or 0.7% agar
BOi2Y	Blaydes (1966) [36]	--	3% sucrose, 0.25% Gelrite or 0.7% agar
MSO	Murashige and Skoog basal salt mixture (1962) [35]		3% sucrose, 0.25% Gelrite or 0.7% agar
